# Identification of key programmed cell death-related genes and immune infiltration in extracorporeal membrane oxygenation treatment for acute myocardial infarction based on bioinformatics analysis

**DOI:** 10.3389/fcvm.2022.1018662

**Published:** 2022-12-02

**Authors:** Jingqi Yang, Xiaochao Ouyang, Ming Yang, Guobo Xie, Qianqiang Cao

**Affiliations:** Department of Cardiovascular Medicine, Jiangxi Provincial People’s Hospital, The First Affiliated Hospital of Nanchang Medical College, Nanchang, China

**Keywords:** extracorporeal membrane oxygenation, acute myocardial infarction, programmed cell death, immune infiltration, bioinformatics

## Abstract

**Background:**

Extracorporeal membrane oxygenation (ECMO) is an important clinical treatment for acute myocardial infarction (AMI) combined with cardiogenic shock, but the role of programmed cell death (PCD)-related genes in prognostication has not yet been investigated. Therefore, we explored the key prognostic biomarkers and immune infiltration in ECMO treatment in AMI combined with cardiogenic shock.

**Methods:**

The GSE93101 dataset was analyzed from the Gene Expression Omnibus (GEO) database, and the expression levels of PCD-related genes in AMI under ECMO were identified. Differentially expressed PCD-related genes between successful and failed treatment samples were analyzed, and Least absolute shrinkage and selection operator (LASSO) logistic regression and random forest were used to screen PCD-related molecular markers for ECMO treatment in AMI combined with cardiogenic shock. Co-expressed regulatory network and enrichment functions of the hub PCD-related genes were performed. In addition, the single-sample gene set enrichment analysis (ssGSEA) algorithm was used to calculate the immune cell infiltration of the ECMO treatment samples.

**Results:**

A total of 115 differentially expressed genes were identified from the GSE93101 dataset, and 76 genes were associated with PCD. Then, two hub PCD-related genes, Cell division cycle associated 7 (CDCA7), ankyrin repeat and SOCS box containing 13 (ASB13) were identified as prognostic markers of ECMO treatment in AMI combined with cardiogenic shock. The most significant Gene Ontology (GO) enriched terms of the co-expressed protein of ASB13 are related to post-translational protein modification, cullin-RING ubiquitin ligase complex, and cullin family protein binding, and the Kyoto Encyclopedia of Genes and Genomes (KEGG) analysis showed that ubiquitin mediated proteolysis is the most enriched pathway. The results of GO and KEGG analysis in CDCA7 were mainly involved in DNA and cell cycle related activities and pathways. Moreover, we found that the successful treatment samples contained a lower proportion of nature killer T cells using immune infiltration analysis. Immune cell infiltration analysis revealed that ASB13 was positively correlated with natural killer cell (*r* = 0.591, *p* = 0.026), monocyte (*r* = 0.586, *p* = 0.028), and gamma delta T cell (*r* = 0.562, *p* = 0.036).

**Conclusion:**

The results of this study showed that ASB13 and CDCA7 may contribute to the occurrence and progression of AMI with cardiogenic shock under ECMO.

## Introduction

Although percutaneous coronary intervention (PCI) has progressed and is widely used, some patients are prone to complications, such as acute heart failure, cardiogenic shock, malignant arrhythmias, and even cardiac arrest after acute myocardial infarction (AMI) (1). Cardiogenic shock remains the leading cause of death in patients with AMI (2). When the patient suffers from cardiogenic shock, the circulatory system is disordered, the left ventricular contractility deteriorates, coronary perfusion is further reduced, and the heart and vital organs continue to be damaged, thus forming a vicious cycle (3).

Among the various processes that occur during myocardial infarction, the programmed cell death (PCD) and immune response play an important role. Programmed cell death, including ferroptosis, necroptosis, apoptosis, and autophagy, was required for maintaining tissue homeostasis in cardiomyocytes, and its mis-regulation was clearly associated with myocardial infarction (4, 5). After myocardial infarction, necrotic cells release danger signals, activate innate immune pathways and trigger a strong inflammatory response, significantly inhibit the process of autophagy, and promote the apoptosis of cardiomyocytes in the infarct border zone (6). However, activation of innate immune pathways in cardiomyocytes produces cytoprotective and pro-survival effects through mitochondrial stabilization, whereas activation that is more prolonged or of greater magnitude and involves immune cells results in more robust inflammatory responses and leukocyte recruitment, which aggravate myocardial injury (7, 8).

Although early revascularization can re-establish blood flow to the coronary arteries and improve myocardial perfusion, it is difficult to correct cardiac function and the shock state immediately. Mechanical circulatory support devices can temporarily replace the heart to provide power for circulation and is an effective auxiliary treatment method. Importantly, extracorporeal membrane oxygenation (ECMO) can significantly improve the cardiac output of patients and is gradually being used in high-risk acute coronary syndrome (ACS) with cardiogenic shock (9, 10). ECMO can pump blood into the extracorporeal membrane lung device for adequate oxygenation and removal of carbon dioxide to ensure effective blood supply (11). Despite important advances in the application of ECMO in patients with AMI and cardiogenic shock, complications and mortality remain high. Resent studies have found that ECMO can affect the immune system through a variety of mechanisms, such as inducing endothelial dysfunction and activating neutrophils, platelets, and blood coagulation pathways (12, 13). Because ECMO therapy may cause immune alterations and usually has uncertain effects, in clinical practice, it is important to identify high-risk patients with acute coronary syndromes who most likely to benefit from the treatment, while also avoiding excessive waste of medical resources.

At present, it is difficult to predict the outcome of ECMO in patients with AMI complicated with cardiogenic shock. With the rapid development of high-throughput sequencing technology, there has been a data explosion in the field of biomedicine, and it has shown great advantages in screening disease biomarkers and therapeutic targets. Through bioinformatics technology, prognostic genes or potential biomarkers of cardiovascular disease can be analyzed (14, 15). Biomarkers may be the key to individualized treatment of ECMO. In AMI combined with cardiogenic shock, we analyzed the peripheral blood mononuclear cells (PBMCs) of patients with successful and failed treatment with ECMO, obtained PCD-related molecular markers, calculated their immune cell composition using the single-sample gene set enrichment analysis (ssGSEA) method (16), and further analyzed the immune cell infiltration and the relevance between immune cells and patient outcome. This study may provide a molecular theoretical basis for the development of PCD biomarkers in ECMO treatment of AMI complicated with cardiogenic shock.

## Materials and methods

### Data processing

The GSE93101 dataset used in the present study was obtained from the Gene Expression Omnibus (GEO) database.^1^ The GSE93101 dataset, based on the GPL14951 platform (Illumina Human V4.0 R2 expression beadchip), included 33 patients with cardiogenic shock; 17 of them survived more than 7 days after ECMO therapy, and 16 died or had multiple organ failure within 7 days. The expression of the whole genome of PBMCs collected from the ECMO device was detected. We extracted the patients’ data from this dataset for analysis, including seven patients who survived more than 7 days (successful treatment group) and seven patients who died or experienced multiple organ failure within 7 days (failure treatment group).

### Programmed cell death-related differential gene screening

We performed base two logarithm conversions, background correction, and quantile normalization on the expression profiles using the “limma” package in R 4.0.4 software (17). After preprocessing, genes with *P* < 0.05 and at least a 0.5-fold change were considered differential genes between the successful and failure treatment groups by the “limma” package (17). The differentially expressed genes were visualized by Heatmap and volcano plot (18).

The list of PCD-related genes were obtained from the GeneCards,^2^ FerrDb,^3^ and the Human Autophagy Database.^4^ In GeneCards, we obtained 13,524 programmed death-related genes, 611 necroptosis-related genes, and 14,395 apoptosis-related genes, by respectively searching programmed death, necroptosis, apoptosis. Furthermore, 428 ferroptosis-related genes and 222 autophagy-related genes respectively from FerrDb Database and Human Autophagy Database. Afterward, we separately intersected the differentially expressed genes with genes related to programmed death, ferroptosis, necroptosis, apoptosis, and autophagy. The online tool jvenn was used to visualize the Venn diagrams (19).

### Screening and verification of hub genes

Least absolute shrinkage and selection operator (LASSO) logistic regression (20), random forest (21), and support vector machine (SVM) (22) algorithms were used to screen PCD-related molecular markers for AMI under ECMO. LASSO was performed according to the “glmnet” package,^5^ and after 10 trials of five-fold cross-validation, the optimal model parameter λ was calculated when the accuracy of the model was the best through 1-standard error (SE) (23). The “Randomforest” package (24) was used to build a random forest model. The model generates classification trees randomly and scores the classification results. Then the model will perform statistical analysis on the classification results of all single trees to obtain high-accuracy classification results. SVM was a supervised machine-learning technique widely used in classification and regression. It can iteratively filter out the feature subset with the highest accuracy rate for a large amount of data. At last, the prognostic molecular markers were obtained by overlapping the prediction results in the three algorithms.

Whole blood samples from ten patients with AMI and cardiogenic shock treated with ECMO were collected for real-time quantitative polymerase chain reaction (qPCR) to confirm the results. Among them, five patients survived more than 7 days after ECMO therapy, and five died within 7 days. Relevant clinical information of the 10 patients was presented in Supplementary material. The study was approved by the Ethics Committee of Jiangxi Provincial People’s Hospital, and all patients signed the informed consent. All patient samples were processed to isolate PBMCs immediately after collection and stored at −80°C before RNA extraction. After the samples were pretreated, RNA was extracted using TRIzol reagent (Invitrogen, Waltham, MA, USA), and qPCR was performed. Relative gene expression was analyzed by the 2^–ΔΔCT^ method with the normalization to ACTB (internal reference gene). All primers used in this study are shown in Supplementary material.

### Construction of co-expressed regulatory network of the hub genes and functional annotation

Co-expressed regulatory network of differentially expressed PCD-related genes was analyzed using STRING database^6^ (25), and the co-expressed regulatory proteins were collected with a moderate confidence >0.4 (26) and constructed a protein-protein interaction (PPI) network using Cytoscape software (v3.8.2) (27). Subsequently, Gene Ontology (GO) (28, 29) and Kyoto Encyclopedia of Genes and Genomes (KEGG) (30, 31) pathway enrichment analyses were performed on PCD-related genes using the package “clusterProfiler” in R (32). The KEGG pathways and GO terms were exhibited according to the “GOplot” package (28), and the significance threshold was set to *P* < 0.05.

### Single-sample gene set enrichment analysis and cell infiltration analysis

The ssGSEA method was used to calculate enrichment scores for 28 immune-related cell types in the samples. The level of immune cell infiltration in each sample were calculated by the ssGSEA function of the GSVA package in R 4.0.4 software (16). We analyzed differences in immune cell infiltration between successful and failure treatment group. Correlations between genes in immune cells and PCD-related hub genes were assessed using Pearson correlation.

## Results

### Differentially expressed immune-related gene screening

**FIGURE 1:**
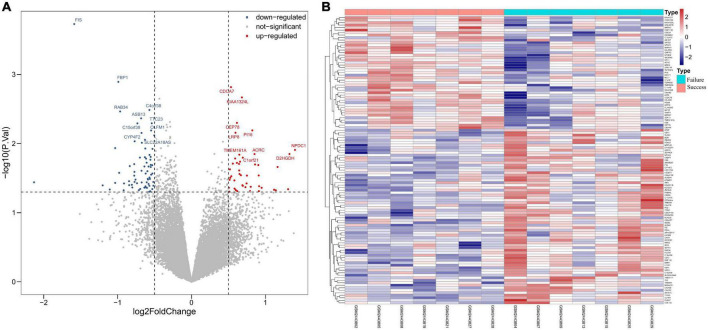
Analysis of programmed cell death (PCD)-related genes in extracorporeal membrane oxygenation (ECMO) treatment for acute myocardial infarction (AMI). **(A)** Volcano plot representing PCD-related genes. In the volcano plots, the red points show upregulated genes (log_2_FC ≥ 0.5 and *P*-value < 0.05), whereas the blue points represent downregulated genes. **(B)** Heatmap of PCD-related differentially expressed genes. The color intensity (from red to blue) suggests higher to lower expression.

The GSE93101 dataset was obtained from the GEO database, and the general clinical information was presented in Table 1. After data processing, a total of 20,297 genes were obtained. Compared with the failure treatment group, a total of 45 genes were up-regulated and 70 were down-regulated in the successful treatment group. The volcanic diagram for the PCD-related genes and the expression heatmap of the PCD-related genes are shown in Figure 1.

**TABLE 1:** Thegeneral clinical information of GSE93101 data set.

Sample	Age	Gender	Group
GSM2443802	47.8	Male	Successful
GSM2443804	67.3	Male	Failure
GSM2443805	52.8	Male	Successful
GSM2443807	78.9	Male	Failure
GSM2443808	53.2	Male	Successful
GSM2443809	70.9	Male	Failure
GSM2443813	52.4	Male	Failure
GSM2443815	52.8	Male	Failure
GSM2443819	57.3	Female	Successful
GSM2443821	49.3	Male	Successful
GSM2443825	63	Male	Failure
GSM2443827	53.6	Male	Successful
GSM2443828	50.1	Female	Successful
GSM2443829	37.4	Male	Failure

### Screening the programmed cell death-related biomarkers

**FIGURE 2:**
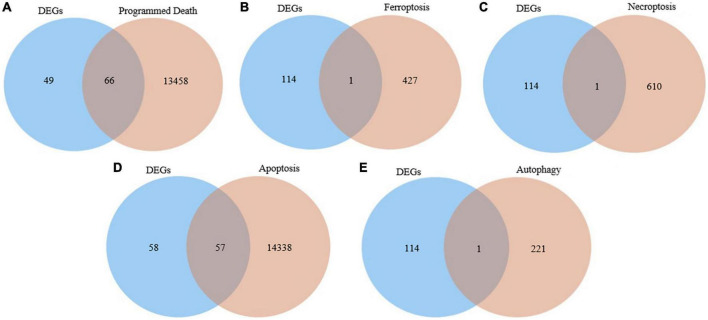
Identification of the candidate programmed cell death (PCD)-related differentially expressed genes. **(A)** Venn diagram to identify common genes between differentially expressed genes and programmed death related genes. **(B)** Venn diagram to identify common genes between differentially expressed genes and ferroptosis related genes. **(C)** Venn diagram to identify common genes between differentially expressed genes and necroptosis related genes. **(D)** Venn diagram to identify common genes between differentially expressed genes and apoptosis related genes. **(E)** Venn diagram to identify common genes between differentially expressed genes and autophagy related genes.

We interacted the 115 differentially expressed genes with 13,524 programmed death-related genes in GeneCards, resulting in 66 common genes (Figure 2A). In addition, one ferroptosis-related gene (Figure 2B), one necroptosis-related gene (Figure 2C), 57 apoptosis-related (Figure 2D), and one autophagy-related gene (Figure 2E) were respectively obtained after the intersection. Finally, we obtained 73 differentially expressed genes related to PCD for further analysis.

### Identification of the programmed cell death-related genes

**FIGURE 3:**
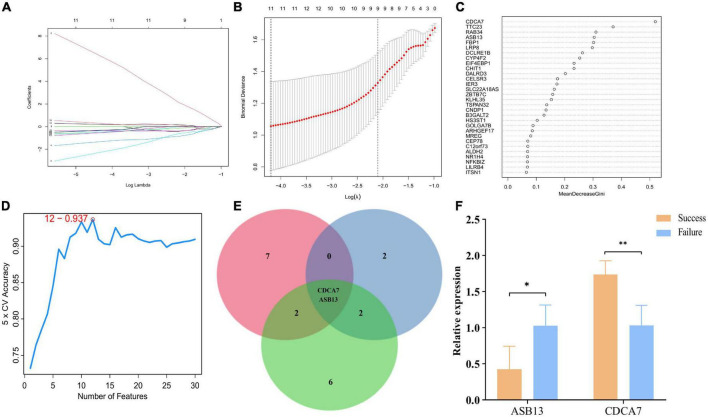
Screening process of programmed cell death (PCD)-related prognostic genes. **(A)** Least absolute shrinkage and selection operator (LASSO) algorithm to screen PCD-related prognostic genes. **(B)** 10-fold cross-validation for the coefficients of 77 PCD-related differentially expressed genes in the LASSO model. **(C)** Plot of top 30 biomarker selected by random forest. **(D)** Further screening of top 30 biomarker by support vector machine (SVM)-RFE algorithm. **(E)** Venn diagrams of hub PCD-related prognostic genes from the three algorithms. **(F)** Quantitative polymerase chain reaction (qPCR) validation of the hub PCD-related prognostic genes; ^*^p < 0.05 vs. the failure treatment group; ^**^p < 0.01 vs. the failure treatment group.

Least absolute shrinkage and selection operator, random forest, and SVM were utilized to explore the candidate PCD-related genes in AMI associated with cardiogenic shock under ECMO. Eleven, six, and twelve PCD-related genes were identified with LASSO (Figures 3A, B), random forest (Figure 3C), and SVM (Figure 3D), respectively. Two hub genes, Cell division cycle associated 7 (CDCA7), ankyrin repeat and SOCS box containing 13 (ASB13), were identified by overlapping the prediction results in the three algorithms (Figure 3E). In addition, the qPCR results showed that in the successful treatment group, the expression of CDCA7 was significantly increased (Figure 3F), while the expression of ASB13 was significantly down-regulated (*P* < 0.01) (Figure 3F). These results were consistent with the results of the microarray data analysis, suggesting that the results were reliable.

### Construction of co-expressed regulatory network and enrichment analyses

**FIGURE 4:**
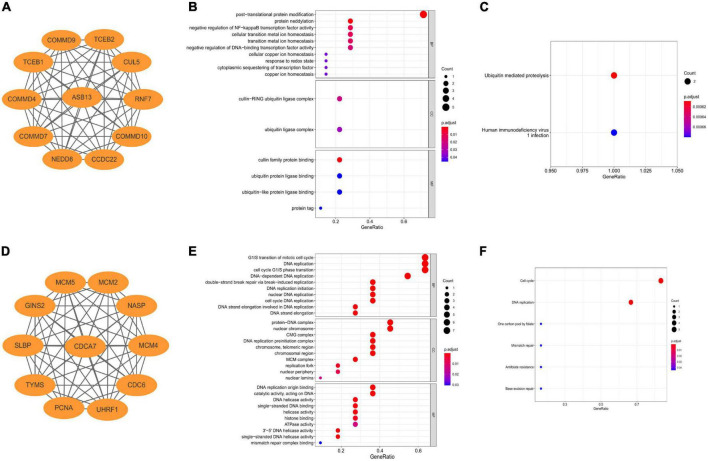
Construction of co-expression regulatory network and enrichment analysis of hub programmed cell death (PCD)-related genes. **(A)** Co-expression regulatory network of ankyrin repeat and SOCS box containing 13 (ASB13). **(B)** Gene ontology (GO) analysis of the regulatory network of ASB13. **(C)** Kyoto Encyclopedia of Genes and Genomes (KEGG) analysis of the regulatory network of ASB13. **(D)** Co-expression regulatory network of cell division cycle associated 7 (CDCA7). **(E)** GO analysis of the regulatory network of CDCA7. **(F)** KEGG analysis of the regulatory network of CDCA7.

The co-expressed protein regulatory network of the two hub PCD-related genes were constructed using the STRING database with a moderate confidence >0.4 (Figures 4A, D) (25). GO and KEGG enrichment analyses were also performed using the co-expressed proteins. The result indicated that the most significant GO enriched terms of the co-expressed protein of ASB13 are related to post-translational protein modification (Biological Process), cullin-RING ubiquitin ligase complex (Cellular Component), and cullin family protein binding (Molecular Function) (Figure 4B). And the KEGG analysis showed that ubiquitin mediated proteolysis was the most enriched pathway (Figure 4C). Moreover, the results of GO and KEGG in CDCA7 related to DNA and cell cycle related activities and pathways (Figures 4E, F).

### Single-sample gene set enrichment analysis and cell infiltration analysis

**FIGURE 5:**
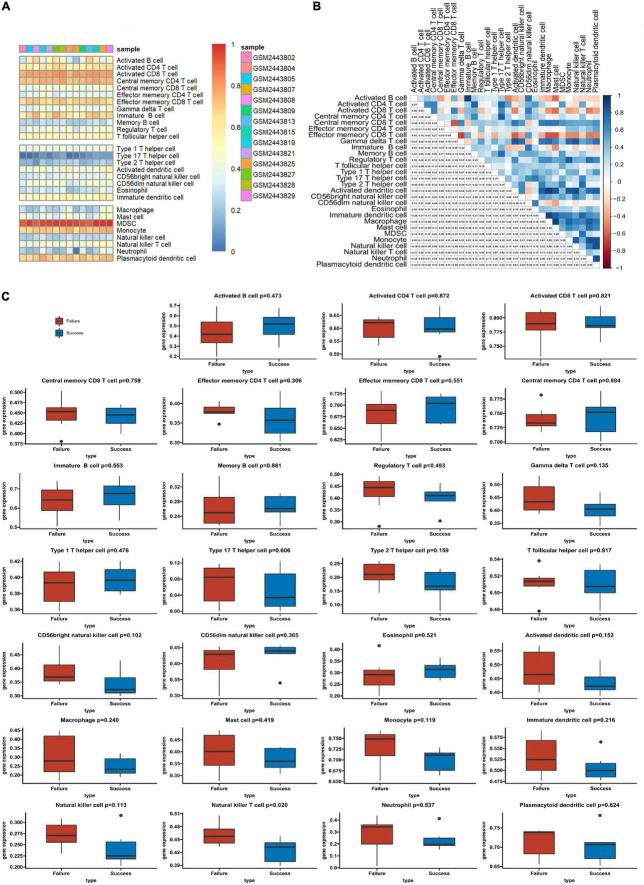
The profiles of immune cell subtype distribution patterns in the GSE93101 dataset. **(A)** Bar plot visualizing the relative percentage of 28 immune cells in each sample. **(B)** Heatmap plot of the correlation between 28 immune cells; blue and red represent positive and negative correlations, respectively. **(C)** Histogram of all 28 differentially infiltrated immune cell fractions.

We used ssGSEA to analyze the abundance of immune cells in the GSE93101 dataset (Figure 5A). The correlation analysis of various immune cells indicated that the positive correlation between immature B cell and activated B cell was the strongest and the negative correlation were observed between effector memory CD8 T cell and central memory CD8 T cell (Figure 5B). The immune infiltration findings indicated that, compared with the failure treatment group, the successful treatment group contained a lower proportion of nature killer T cells (Figure 5C).

### Correlation analysis between the two programmed cell death-related genes and infiltrating immune cells

**FIGURE 6:**
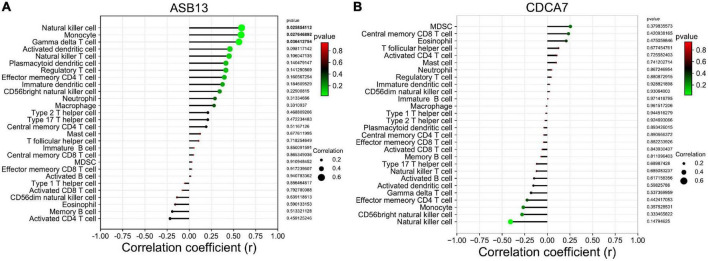
Correlation between hub programmed cell death (PCD)-related prognostic genes and infiltrating immune cells in extracorporeal membrane oxygenation (ECMO) treatment for acute myocardial infarction (AMI). **(A)** Immune correlation analysis of ankyrin repeat and SOCS box containing 13 (ASB13). **(B)** Immune correlation analysis of cell division cycle associated 7 (CDCA7).

The results of the correlation analysis revealed that ASB13 was positively correlated with natural killer cell (*r* = 0.591, *p* = 0.026), monocyte (*r* = 0.586, *p* = 0.028), and gamma delta T cell (*r* = 0.562, *p* = 0.036) (Figure 6A). However, it seems that CDCA7 has no obvious correlation with the immune cells (Figure 6B).

## Discussion

Cardiogenic shock is a state of low cardiac output associated with hypotension and end-organ hypoperfusion (33). The initiation of ECMO can oxygenate venous blood outside the body and partially replace the body’s heart and lung function to maintain blood and oxygen perfusion, and this has emerged as a salvage intervention (34, 35). Regardless of the occurrence of cardiogenic shock after AMI or the use of ECMO, it stimulates immune and inflammatory responses (36, 37), and PCD is essential for the development of the immune system and maintaining the response to exogenous and endogenous stimuli (38). Therefore, the identification of PCD-related molecular mechanisms and prognostic genes can effectively identify which patient can benefit from ECMO, in AMI complicated with cardiogenic shock.

In the GSE93101 dataset, a total of 75 differentially expressed PCD-related genes were detected as candidate biomarkers for the prognosis of ECMO treatment in AMI associated with cardiogenic shock. Then, according to the LASSO, random forest, and SVM algorithms, ASB13 and CDCA7 were selected as the hub PCD-related genes, and the underlying mechanism was determined through the enrichment function. Finally, we analyzed the abundance of immune cells and the relationship between the hub PCD-related genes and immune cell infiltrations.

Apoptosis is a type of PCD (39). Cardiomyocyte apoptosis is the main pathological mechanism leading to heart failure and cardiogenic shock in myocardial infarction, and mitochondria-mediated internal apoptosis pathway is the main reason for the induction of cardiomyocyte apoptosis (5). Mitochondria are abundant in cardiomyocytes, and the metabolism provides 90% of the adenosine triphosphate (ATP) required for cardiac contractile activity. The integrity of mitochondrial structure and function is the premise and basis of normal cell metabolism, and mitochondria can also regulate intracellular ion balance, and then participate in the processes of cell apoptosis and necrosis (40). In the animal model of ventricular fibrillation-induced cardiac arrest, ECMO can prevent oxidative damage, regulate energy metabolism, inhibit cardiomyocyte apoptosis, and improve survival (41). We also found that, the two hub genes, ASB13 and CDCA7, were both apoptosis-related genes, suggesting that ECMO may affect the prognosis of patients through apoptosis, in the AMI complicated by cardiogenic shock.

Ankyrin repeat and SOCS box containing 13, is a member of the ASB protein family, and contain ankyrin repeat sequence and belongs to the SOCS box protein superfamily (42). The ankyrin motif repeat domain at the N-terminus of ASB13 consists of six ankyrins, which can mediate the interaction of various ASB proteins with other proteins in different functions, so that each ASB protein participates in different cellular biological processes (43). The C-terminal SOCS box domain can be divided into two subdomains: BC box and Cul5 box. The BC box subdomain can bind to the proteins Elongin B and Elongin C and the Cul5 box can bind to the N-terminus of the Cullin5 protein. The C-terminus of Cullin5 can recruit Rbx2 protein to form the ASB13-Elongin B/C-Cul5-Rbx2 complex, while Rbx2 can bind to E2 ubiquitin-conjugating enzyme bound to ubiquitin protein (44). However, the ubiquitin system is complex, multifaceted, and essential for the regulation of numerous cellular processes (45). Post-translational modification of proteins by ubiquitin regulates many steps of the autophagy and cell death pathways (46). The relationship between ASB13 and AMI or ECMO treatment has not been directly reported in the literature. ECMO may also affect human PCD-related cells, so further research is needed.

Cell division cycle associated 7 (CDCA7), was a novel c-Myc response gene, located on human chromosome 2q31, encoding the nuclear protein composed of 371 amino acids (47). CDCA7 was periodically expressed during the cell cycle, reaching its highest level between G1 and S phases. The study found that CDCA7 was associated with Myc, and this association was regulated in a phosphorylation-dependent manner (48). Studies have shown that CDCA7 was a downstream target gene of transcription factor Myc and E2F transcription factor 1, participates in cell cycle process, and can be used as a transcriptional regulator of expression (49). This suggests that CDCA7 may regulate cell proliferation or apoptosis by regulating the expression of some genes, and participate in the occurrence and development of PCD. In addition, in the absence of CDCA7, proteins involved in maintaining DNA methylation were significantly reduced on nascent DNA (50). Therefore, CDCA7 plays an important role in the metabolism. This also further supports our results, in patients with AMI and cardiogenic shock under ECMO, the expression level of CDCA7 in the successful treatment group was significantly upregulated.

The types of immune cell infiltration in the successful and failure treatment groups were analyzed by ssGSEA. The nature killer T cells were found to be potentially related to the outcome of cardiogenic shock under ECMO in AMI. Correlation analysis between the hub PCD-related genes and immune cells found that ASB13 was positively correlated with natural killer cell, monocyte and gamma delta T cell. It is well known that the strong immune and inflammatory responses after AMI are related to cardiac remodeling and myocardial recovery. However, the initiation of ECMO in patients with AMI also causes complex immune and inflammatory reactions. Sustained immune responses result in endothelial injury, leukocyte activation, and the production of proinflammatory mediators, and it is unclear whether this excessive immunity has potential benefits or is deleterious to patients (37).

Our study has some limitations. On the one hand, the number of cases in the GSE93101 dataset was relatively small, and the hub PCD-related genes obtained by the three algorithms used were not verified by more experiments. On the other hand, the study is descriptive, and further molecular experiments are needed to validate the data.

In summary, this was the first study to predict PCD-related genes biomarkers in patients with AMI combined with cardiogenic shock under ECMO. We found that ASB13 and CDCA7 may contribute to the occurrence and progression of AMI with cardiogenic shock under ECMO. Moreover, the correlations between the PCD-related genes and immune cells may play a significant role in the pathogenesis of AMI combined with cardiogenic shock under ECMO. These findings enhance our understanding of the molecular mechanisms in AMI under ECMO, although the exact molecular mechanism and functional pathways warrant further exploration.

## Data availability statement

Publicly available datasets were analyzed in this study. This data can be found here: https://www.ncbi.nlm.nih.gov/geo/query/acc.cgi?acc=GSE93101.

## Ethics statement

The studies involving human participants were reviewed and approved by the Ethics Committee of Jiangxi Provincial People’s Hospital. The patients/participants provided their written informed consent to participate in this study.

## Author contributions

MY and JY: conception and design. XO: administrative support. GX: collection and assembly of data. QC and MY: data analysis and interpretation. JY, QC, and MY: manuscript writing. All authors contributed to the article and approved the submitted version.

## Footnotes

1
http://www.ncbi.nlm.nih.gov/geo/

2
https://www.genecards.org/

3
http://www.zhounan.org/ferrdb/

4
http://www.autophagy.lu/

5
https://CRAN.R-project.org/package=glmnet

6
http://string-db.org
